# Feedback from physical activity monitors to enhance amount of physical activity in adults—a protocol for a systematic review and meta-analysis

**DOI:** 10.1186/s13643-019-0970-3

**Published:** 2019-02-12

**Authors:** Rasmus Tolstrup Larsen, Vibeke Wagner, Camilla Keller, Carsten Bogh Juhl, Henning Langberg, Jan Christensen

**Affiliations:** 1 CopenRehab, Department of Public Health, Section of Social Medicine, University of Copenhagen, Gothersgade 160, 3rd floor, 1123 Copenhagen K, Denmark; 2 Department of Neurorehabilitation/TBI Unit, Copenhagen University Hospital, Denmark (Hvidovre Hospital), Copenhagen, Denmark; 3 Research Unit of Musculoskeletal Function and Physiotherapy, Institute of Sports Science and Clinical Biomechanics, Faculty of Health Sciences, University of Southern Denmark, Odense, Denmark; 4 Department of Physiotherapy and Occupational Therapy, Copenhagen University Hospital, Herlev, Gentofte, Denmark; 5 Department of Occupational- and Physiotherapy, Copenhagen University Hospital, Copenhagen, Denmark; 6 National Centre for Rehabilitation and Palliative Care, University of Southern Denmark and Odense University Hospital, Odense, Denmark

## Abstract

**Objective:**

The use of physical activity monitors (PAMs) in the adult population is increasing rapidly and previous systematic reviews are outdated. The objective of this systematic review and meta-analysis is to estimate the effect of PAM-based interventions on physical activity behavior in adults. Furthermore, the impact of intervention, study, and participants characteristics will be investigated.

**Methods and design:**

Randomized controlled trials and randomized cross-over trials including adult participants will be included if the study compares any PAM-based intervention where the participants receive feedback on their physical activity level measured by PAMs, to control interventions where participants do not receive feedback from PAMs. This protocol is detailed according to the recommendations of the Cochrane Handbook, and it is reported according to the preferred reporting items for systematic reviews and meta-analyses protocols statement. The results from the literature search will be presented in a PRISMA flow chart. The effects from individual studies will be summarized in a random effects meta-analysis and the impact of diagnosis of the participants, type of feedback, type of intervention, and control intervention will be investigated in stratified meta-analysis and meta-regressions analysis. The results on daily physical activity, moderate to vigorous physical activity, sedentary time, and adverse events will be presented in a summary of findings table.

**Discussion:**

The results will be useful to researchers, policy makers, and health care professionals when the intention is to increase physical activity in the adult population.

**PROSPERO registration:**

CRD42018102719

**Electronic supplementary material:**

The online version of this article (10.1186/s13643-019-0970-3) contains supplementary material, which is available to authorized users.

## Background

### Rationale

Physical activity is defined as any bodily movement that requires energy expenditure [1]. According to the World Health Organization (WHO), one in four adults does not meet the recommendations for physical activity and physical inactivity is estimated to be responsible for 9% of the world’s premature deaths [2]. Globally, inactivity costs 54 billion USD in direct health care cost every year [1]. Furthermore, strong evidence exists that physically active people will have reduced rates of all-cause mortality, coronary heart disease, high blood pressure, stroke, metabolic syndrome, type 2 diabetes, breast cancer, colon cancer, depression, and falls [2]. A global plan from the WHO focused on reducing the relative prevalence of physical inactivity by 15% in 2030. In order to progress towards this goal WHO states that effective community-based interventions are crucial [1].

Physical activity monitors (PAMs) have been found to be an effective facilitator to increase physical activity [3] and reduce sedentary time [4] and have a positive influence on weight loss among individuals in weight loss programs [5]. Furthermore, the feedback from PAMs are suggested to motivate behavioral change [6], and therefore, PAM-based interventions hold great potential for increasing physical activity. PAMs were originally designed to quantify the level of physical activity [4] and have been used in research since the 1960s [5]. Due to the technological development, the accessibility ,and relatively low cost of PAMs, they are incorporated in many devices and could potentially be one of the keys to effective large-scale community-based interventions and thereby increasing physical activity on a global level [1]. Earlier systematic reviews and meta-analyses have shown that pedometers can increase physical activity among outpatient adults [7] and reduce sedentary time in adults [4]. However, there are two reasons for performing a new systematic review and meta-analysis on the effect of PAM-based interventions on physical activity. Firstly, since recent systematic reviews were conducted, technology has improved rapidly and many of today’s PAMs can monitor number of steps taken and provide real-time feedback on several measures. As a result of this, the amount of scientific papers investigating PAMs impact on physical activity has increased equivalent. Secondly, with the increasing body of evidence, it might be possible to do further subgroup analyses investigating which study and participant characteristics that can explain effect size heterogeneity in the literature.

An update of the body of evidence regarding PAMs effect on daily physical activity, moderate to vigorous physical activity, and time spent sedentary with an investigation of the intervention, study, and participants characteristics is deemed necessary in order to progress towards WHO’s declared goal to reduce the prevalence of physical inactivity [1], to inform future studies, and to inform clinical guidelines.

### Objective

The objective of this systematic review and meta-analysis is to estimate the effect on daily physical activity, moderate to vigorous physical activity, and time spent sedentary when using feedback from PAMs in interventions, compared to control interventions where the participants do not receive feedback from PAMs, in participants aged 18–64. Subsequently, the impact of intervention, study, and participants characteristics on the effect of PAM will be investigated.

## Methods

This protocol is detailed according to the recommendations of the Cochrane Handbook [8] and it is reported according to the preferred reporting items for systematic reviews and meta-analyses protocols (PRISMA) statement [9]. This protocol has been registered in the PROSPERO database with the registration number CRD42018102719.

### Eligibility criteria

#### Types of studies

Randomized controlled trials and randomized cross-over trials will be included.

#### Types of participants

For studies to be eligible for inclusion in this systematic review and meta-analysis, 80% of the study participants must be above 18 years of age and below 65 years of age. Age distribution are calculated from the mean and SD, as described by Hall et al. [10].

#### Types of interventions

Studies comparing any PAM-based intervention where the participants received feedback on their physical activity level measured by PAMs will be included. Feedback is defined as any result on physical activity, measured objectively by the PAMs including but not limited to inactivity notifications. The PAMs may be portable or wearable, electronic or mechanical, and driven by accelerometers, pedometers, or global positioning system (GPS).

#### Types of comparators

In all control interventions, the participants cannot receive any kind of feedback on their physical activity level from PAMs. The participants of control interventions can wear PAMs, but if so, the PAMs should be sealed and all feedback should be disabled.

#### Types of outcome measures

The three primary outcomes are the following:

1. Change in daily physical activity

If more than one relevant outcome is reported within a study, the outcome will be extracted or calculated favoring daily number of steps, followed by daily number of meters walked, daily amount of energy expenditure measured as calories, daily metabolic equivalent of task (minutes or hours), and finally, if no objective measure is available, self-reported physical activity.

2. Change in moderate to vigorous physical activity

If more than one relevant outcome is reported within a study, the outcome will be extracted or calculated favoring objectively measured moderate to vigorous physical activity, followed by self-reported moderate to vigorous physical activity. If more than one type of moderate to vigorous physical activity (MVPA) classification is used on accelerometry data, the study-specific primary classification will be extracted from the study.

3. Change in sedentary time

If more than one relevant outcome is reported within a study, the outcome will be extracted or calculated favoring objectively measured sedentary time, followed by self-reported sedentary time.

### Adverse events

Reported adverse events and drop-outs will be extracted.

### Timing and effect measures

Data will be extracted at post-intervention and follow-up.

### Search methods for identification of studies

An electronic search for eligible studies in the electronic databases MEDLINE, EMBASE, SPORTDiscus, CINAHL, and Cochrane Central Register of Controlled Trials (CENTRAL) was conducted in July 2018, according to the PROSPERO protocol.

The search matrix illustrated in Table 1 will consist of a combination of relevant keywords and MeSH/Thesaurus terms for (1) PAMs and (2) study design.

**Table 1:** Search matrix for electronic searches

Physical activity monitoring	Study design
pam AND monitor*	“randomly”
physic* AND activit* AND monitor*	“randomized controlled trial”
“activity monitoring device”	“controlled clinical trial”
“fitness tracker*”	“cross-over trial”
“quantified movement”	“cross over trial”
“movement counter*”	“randomized”
“jawbone”	“clinical trial”
“vivoactive”	
“tomtom”	
“xiaomi mi band”	
“accelerometer-based tracker*”	
“moov now”	
“misfit ray”	
“nokia go”	
“activity monitor*”	
fitbit	
pedometer*	
“step monitor*”	
“physical activity monitor*”	
“Step counter*”	
actigraph	
Gt3x	
wGT3X-BT	
GT9X	
axivity	
acceleromet*	

*Database specific subject headings related to study type will be added*

*Database-specific subject headings related to physical activity monitoring will be added*

No restrictions on language or publication-time will be applied. If relevant studies are identified in other language than Danish, English, Swedish, Norwegian, and German, a relevant translator will be contacted. The authors of unobtainable studies or studies with missing data will be contacted.

The detailed search strategy for each database can be found in the Additional file 1.

### Searching other resources

Searching references of eligible studies and relevant journals (pearl growing) will be conducted independently by two reviewers (CK and VW) in order to include relevant articles not captured by the search strings. The database ClinicalTrials.gov will be searched to locate ongoing studies.

### Data collection and analysis

The technology platform, Covidence (Covidence systematic review software, Veritas Health Innovation, Melbourne, Australia. Available at www.covidence.org), will be used to import citations from the literature searches, screening of title and abstracts, screening of full text, assessing risk of bias in included studies, and extracting the data. The analyses will be conducted in Stata Statistical Software, version 15.

### Selection of studies

The selection of studies will be done by merging search results from the databases and removing duplicates. Two review authors (CK and VW) will then independently screen titles and abstracts. Articles judged as eligible by at least one of the reviewers will be screened in independently full-text by the same review authors (CK and VW). Any inconsistencies between authors will be discussed and solved with consultation of a third author (RTL).

### Data extraction and management

Data on the following items will be extracted from all included studies.

Source: Study ID, protocol ID, review author, citation details and contact details.

#### Methods

Study design, aim of study, number of arms or groups, funding source, informed consent obtained, ethical approval.

#### Participants

Total number of participants, setting, possible diagnostic criteria, age, sex, country, co-morbidities, education length, and marriage status.

#### Interventions

Duration of intervention, specific intervention and intervention details sufficient for replication.

#### Outcomes

All outcomes specified in the ‘types of outcome measures’ and specific time points, outcome definitions and unit of measurement.

#### Results

Number of participants allocated to each group, summary data for each intervention and control groups including adverse events and drop outs.

#### Miscellaneous.

Funding sources, key conclusions, miscellaneous comments from authors and if correspondence was required.

### Risk of bias assessment

Two review authors (VW and CK) will independently assess the risk of bias in included studies, using the RoB 2.0 tool [11]. Disagreement between reviews authors will be solved by including a third reviewer (RTL). The risk of bias assessment for each study will be presented using a table with judgment and support for judgment.

### Strategy for data synthesis

The effect size will be calculated as a standardized mean difference of the final scores using a random-effects meta-analysis adjusting to Hedges’ *g*. If only one study is included on an outcome and a meta-analysis is not possible to conduct, a narrative review on the specific outcome will be performed.

If a study has more than one intervention group relevant for inclusion in the systematic review, the intervention groups will be included as two separate studies and the control group will be separated.

Treatment effect, measured as continuous data, will be expressed as mean difference with 95% confidence intervals for outcomes measured with the same outcome measurement instrument or as standardized mean difference with 95% confidence intervals, when different measurement instruments are used in included the studies. Dichotomous outcomes, such as adverse events and drop-outs, will be analyzed and expressed as risk ratios with 95% confidence intervals.

The heterogeneity of the results will be examined using Cochrane Q test and quantified as *I*^2^ values and the between study variance *τ*^2^. Assessment of small study bias will be done by calculating an Egger’s test score and illustrated with a funnel plot. If small study bias is found, by a positive Egger’s test, a metatrim analysis will be conducted and an adjusted effect size will be calculated. For all statistical analysis, an alpha level of 0.05 will be considered statistically significant.

### Analysis of heterogeneity

We will explore heterogeneity by conducting sub-group analyses and stratified analyses on the following nominal variables:

- Diagnoses (healthy, cancer patients, pulmonary patients, cardiovascular patients, musculoskeletal patients, neurological patients or psychiatric patients)
- Feedback frequency (daily, weekly or monthly)
- Grouping of intervention content other than PAM feedback (examples: gamification, counseling and motivational talks, visualization of feedback)
- Content of control intervention (active vs non-active control)
- Feedback on meeting the study specific public health recommendations for physical activity e.g. 10,000 steps per day, 30 min of MVPA per day or 150 min of MVPA per week (yes or no)
- Did the participants in the intervention groups receive disease risks on their level of physical activity e.g., with your age and current level of daily activity you have 40.3% chance of developing type 2 diabetes (yes/no)?

We will explore heterogeneity on continuous data by performing univariate meta-regressions on the following variables:

- Mean age
- Sex distribution
- Intervention length
- Body mass index
- Mean baseline physical activity
- Setting and national income per capita (country specific gross domestic product per capita data from the World Bank)

The impact of risk of bias will be analyzed on all outcomes by stratifying on overall risk of bias, defined by the RoB 2.0 tool (low/some concerns/high) [11].

### Summary of findings table

We will create a summary of findings table with effect sizes on all outcomes. Two reviewers (RTL and JC) will independently rate the quality of the body of evidence using the Grades of Recommendation, Assessment, Development and Evaluation (GRADE) approach including downgrading and upgrading rating, which will be described in the footnotes in the table [12, 13].

## Discussion

The aim of this systematic review and meta-analysis is to systematically locate, evaluate, summarize, and analyze available evidence regarding the effect of PAM-based interventions in adults. Furthermore, this systematic review will investigate the impact of participants and interventions characteristics on the results. When implementing technology interventions to facilitate behavioral change, some differences between younger and older populations are expected. Therefore, we have chosen to use the same age spans as the WHO and focus this systematic review on adults below 65 years of age as we have an ongoing review focused on older adults [14, 15].

The discussion will include a strengths and limitations of the systematic review and evaluate the use of PAM-based interventions to decrease the prevalence of physical inactivity globally, as highlighted by the World Health Organization [1]. Furthermore, the results will be of interest for researchers, policy makers, and health care professionals who aim to increase physical activity among the adult population.

### Strength of this systematic review and meta-analysis

This systematic review and meta-analysis will be conducted according to the current recommendations from the Cochrane Handbook and reported according to the PRISMA statement [16, 17]. The methodological quality of this review and the expected large body of evidence should be used to generate trustworthy recommendations regarding the use of PAM-based physical activity interventions. The use of PAMs has already been documented [4, 7], but as innovative health care solutions and health care technology are rapidly growing fields, this systematic review will provide an updated meta-analysis of the use of PAM in interventions to increase physical activity.

## Additional file

Additional file 1: Search strategy. (DOCX 18 kb)

## Abbreviations

CENTRAL: Cochrane Central Register of Controlled Trials
GPS: Global Positioning System
MVPA: Moderate to vigorous physical activity
PAM: Physical Activity Monitor
PRISMA: Preferred Reporting Items for Systematic Reviews and Meta-Analyses
WHO: World Health Organization
