# Supplementary material for: Feedback from physical activity monitors to enhance amount of physical activity in adults—a protocol for a systematic review and meta-analysis
Source: Syst Rev. 2019 Feb 12;8:53. doi: 10.1186/s13643-019-0970-3 (PMC6371423; doi:10.1186/s13643-019-0970-3)
Supplement: Supplementary file 1 — Search strategy. (DOCX 18 kb) [file 13643_2019_970_MOESM1_ESM.docx]

## Appendix

## Search strategy

Medline
Search (((((((((((((((((((((((((((((pam[Title/Abstract] AND monitor*[Title/Abstract])) OR (physic*[Title/Abstract] AND activit*[Title/Abstract] AND monitor*[Title/Abstract])) OR “activity monitoring device”[Title/Abstract]) OR “fitness tracker*”[Title/Abstract]) OR “quantified movement”[Title/Abstract]) OR “movement counter*”[Title/Abstract]) OR “jawbone”[Title/Abstract]) OR “vivoactive”[Title/Abstract]) OR “tomtom”[Title/Abstract]) OR “xiaomi mi band”[Title/Abstract]) OR ”accelerometer-based tracker*”[Title/Abstract]) OR “moov now”[Title/Abstract]) OR “misfit ray”[Title/Abstract]) OR “nokia go”[Title/Abstract]) OR ”activity monitor*”[Title/Abstract]) OR fitbit[Title/Abstract]) OR pedometer*[Title/Abstract]) OR “step monitor*”[Title/Abstract]) OR “physical activity monitor*”[Title/Abstract]) OR “Step counter*”[Title/Abstract]) OR actigraph[Title/Abstract]) OR Gt3x[Title/Abstract]) OR wGT3X-BT[Title/Abstract]) OR GT9X[Title/Abstract]) OR axivity[Title/Abstract]) OR accelerometry[MeSH Major Topic]) OR actigraphy[MeSH Major Topic])) AND ((((“randomized controlled trial”[MeSH Major Topic]) OR randomized controlled trial[Publication Type]) OR random allocation[MeSH Major Topic]) OR (“randomly”[Title/Abstract] OR “clinical trial”[Title/Abstract] OR “randomized”[Title/Abstract] OR “cross over trial”[Title/Abstract] OR “cross-over trial”[Title/Abstract] OR “controlled clinical trial”[Title/Abstract] OR “randomized controlled trial”[Title/Abstract]))

## CENTRAL

ID Search

#1 MeSH descriptor: [accelerometry] explode all trees

#2 ((pam AND monitor*)):ti,ab,kw

#3 ((physic* AND activit* AND monitor*)):ti,ab,kw

#4 (“activity monitoring device”):ti,ab,kw

#5 (“fitness tracker*”):ti,ab,kw

#6 (“quantified movement”):ti,ab,kw

#7 (“movement counter*”):ti,ab,kw

#8 (“jawbone”):ti,ab,kw

#9 (“vivoactive”):ti,ab,kw

#10 (“tomtom”):ti,ab,kw

#11 (“xiaomi mi band”):ti,ab,kw

#12 (”accelerometer-based tracker*”):ti,ab,kw

#13 (“moov now”):ti,ab,kw

#14 (“misfit ray”):ti,ab,kw

#15 (“nokia go”):ti,ab,kw

#16 (”activity monitor*”):ti,ab,kw

#17 (fitbit):ti,ab,kw

#18 (pedometer*):ti,ab,kw

#19 (“step monitor*”):ti,ab,kw

#20 (“physical activity monitor*”):ti,ab,kw

#21 (“Step counter*”):ti,ab,kw

#22 (actigraph):ti,ab,kw

#23 (Gt3x):ti,ab,kw

#24 (wGT3X-BT):ti,ab,kw

#25 (GT9X):ti,ab,kw

#26 (axivity):ti,ab,kw

#27 #1 OR #2 OR #3 OR #4 OR #5 OR #6 OR #7 OR #8 OR #9 OR #10 OR #11 OR #12 OR #13 OR #14 OR #15 OR #16 OR #17 OR #18 OR #19 OR #20 OR #21 OR #22 OR #23 OR #24 OR #25 OR #26

## EMBASE

1. exp accelerometer/ or exp accelerometry/

2. (pam and monitor*).ab,ti.

3. (physic* and activit* and monitor*).ab,ti.

4. 'activity monitoring device'.ab,ti.

5. 'quantified movement'.ab,ti.

6. 'movement counter*'.ab,ti.

7. 'jawbone'.ab,ti.

8. 'vivoactive'.ab,ti.

9. 'tomtom'.ab,ti.

10. 'xiaomi mi band'.ab,ti.

11. 'accelerometer-based tracker*'.ab,ti.

12. 'moov now'.ab,ti.

13. 'misfit ray'.ab,ti.

14. 'nokia go'.ab,ti.

15. 'activity monitor*'.ab,ti.

16. 'fitbit'.ab,ti.

17. 'pedometer*'.ab,ti.

18. 'step monitor*'.ab,ti.

19. 'physical activity monitor*'.ab,ti.

20. 'Step counter*'.ab,ti.

21. 'actigraph'.ab,ti.

22. 'Gt3x'.ab,ti.

23. 'wGT3X-BT'.ab,ti.

24. 'GT9X'.ab,ti.

25. 'axivity'.ab,ti.

26. 'acceleromet*'.ab,ti.

27. 1 or 2 or 3 or 4 or 5 or 6 or 7 or 8 or 9 or 10 or 11 or 12 or 13 or 14 or 15 or 16 or 17 or 18 or 19 or 20 or 21 or 22 or 23 or 24 or 25 or 26

28. controlled clinical trial/ or "randomized controlled trial (topic)"/

29. 'randomly'.ab,ti.

30. 'randomized controlled trial'.ab,ti.

31. 'controlled clinical trial'.ab,ti.

32. 'cross-over trial'.ab,ti.

33. 'cross over trial'.ab,ti.

34. 'randomized'.ab,ti.

35. 'clinical trial'.ab,ti.

36. 28 or 29 or 30 or 31 or 32 or 33 or 34 or 35

37. 27 and 36

## CINAHL

1. TI ((pam AND monitor*) OR (physic* AND activit* AND monitor*) OR (“activity monitoring device”) OR (“fitness tracker*”) OR (“quantified movement”) OR (“movement counter*”) OR (“jawbone”) OR (“vivoactive”) OR (“tomtom”) OR (“xiaomi mi band”) OR (”accelerometer-based tracker*”) OR (“moov now”) OR (“misfit ray”) OR (“nokia go”) OR (”activity monitor*”) OR (fitbit) OR pedometer*) OR (“step monitor*”) OR (“physical activity monitor*”) OR (“step counter*”) OR (actigraph) OR (Gt3x) OR (wGT3X-BT) OR (GT9X) OR (axivity) OR (acceleromet*))
2. AB ((pam AND monitor*) OR (physic* AND activit* AND monitor*) OR (“activity monitoring device”) OR (“fitness tracker*”) OR (“quantified movement”) OR (“movement counter*”) OR (“jawbone”) OR (“vivoactive”) OR (“tomtom”) OR (“xiaomi mi band”) OR (”accelerometer-based tracker*”) OR (“moov now”) OR (“misfit ray”) OR (“nokia go”) OR (”activity monitor*”) OR (fitbit) OR pedometer*) OR (“step monitor*”) OR (“physical activity monitor*”) OR (“step counter*”) OR (actigraph) OR (Gt3x) OR (wGT3X-BT) OR (GT9X) OR (axivity) OR (acceleromet*))
3. MH (accelerometry)
4. 1 OR 2 OR 3
5. TI ((“randomized controlled trial*”) OR (randomized controlled trial) OR (random allocation) OR (“randomly”) OR (“clinical trial”) OR (“randomized”) OR (“cross over trial”) OR (“cross-over trial”) OR (“controlled clinical trial”) )
6. AB ((“randomized controlled trial*”) OR (randomized controlled trial) OR (random allocation) OR (“randomly”) OR (“clinical trial”) OR (“randomized”) OR (“cross over trial”) OR (“cross-over trial”) OR (“controlled clinical trial”) )
7. MH (randomized controlled trials)
8. 5 OR 6 OR 7
9. 4 AND 8

## SportsDISCUS

1. TI ((pam AND monitor*) OR (physic* AND activit* AND monitor*) OR (“activity monitoring device”) OR (“fitness tracker*”) OR (“quantified movement”) OR (“movement counter*”) OR (“jawbone”) OR (“vivoactive”) OR (“tomtom”) OR (“xiaomi mi band”) OR (”accelerometer-based tracker*”) OR (“moov now”) OR (“misfit ray”) OR (“nokia go”) OR (”activity monitor*”) OR (fitbit) OR pedometer*) OR (“step monitor*”) OR (“physical activity monitor*”) OR (“step counter*”) OR (actigraph) OR (Gt3x) OR (wGT3X-BT) OR (GT9X) OR (axivity) OR (acceleromet*))
2. AB ((pam AND monitor*) OR (physic* AND activit* AND monitor*) OR (“activity monitoring device”) OR (“fitness tracker*”) OR (“quantified movement”) OR (“movement counter*”) OR (“jawbone”) OR (“vivoactive”) OR (“tomtom”) OR (“xiaomi mi band”) OR (”accelerometer-based tracker*”) OR (“moov now”) OR (“misfit ray”) OR (“nokia go”) OR (”activity monitor*”) OR (fitbit) OR pedometer*) OR (“step monitor*”) OR (“physical activity monitor*”) OR (“step counter*”) OR (actigraph) OR (Gt3x) OR (wGT3X-BT) OR (GT9X) OR (axivity) OR (acceleromet*))
3. (DE "ACCELEROMETERS" OR DE "SPEEDOMETERS")
4. 1 OR 2 OR 3
5. TI ((“randomized controlled trial*”) OR (randomized controlled trial) OR (random allocation) OR (“randomly”) OR (“clinical trial”) OR (“randomized”) OR (“cross over trial”) OR (“cross-over trial”) OR (“controlled clinical trial”))
6. AB ((“randomized controlled trial*”) OR (randomized controlled trial) OR (random allocation) OR (“randomly”) OR (“clinical trial”) OR (“randomized”) OR (“cross over trial”) OR (“cross-over trial”) OR (“controlled clinical trial”))
7. SU (randomized controlled trials)
8. 5 OR 6 OR 7
9. 4 AND 8
